# Treatment of Radiation Bone Injury with Transplanted hUCB-MSCs via Wnt/*β*-Catenin

**DOI:** 10.1155/2021/5660927

**Published:** 2021-11-28

**Authors:** Yufeng Zhang, Huaxin Deng, Zhiqiang Yang, Zhe Chen, Sheng Zhang, Yufan Zhu, Min Yang, Houcheng Zhong, Fuling Zhou, Yuanlong Xie, Lin Cai

**Affiliations:** ^1^Department of Spine Surgery and Musculoskeletal Tumor, Department of Orthopedics, Zhongnan Hospital of Wuhan University, Wuhan, China; ^2^Department of Spine Surgery, The Central Hospital of Yongzhou, Yongzhou, China; ^3^Department of Haematology, Zhongnan Hospital of Wuhan University, Wuhan, China

## Abstract

Radiation-induced bone injury (RIBI) is one of the complications after radiotherapy for malignant tumors. However, there are no effective measures for the treatment of RIBI in clinical practice, and the mechanism of RIBI is unclear. We use a single high-dose ionizing radiation (6Gy) to analyze the effect of radiotherapy on osteoblast function. Human umbilical cord blood mesenchymal stem cells (hUCB-MSCs) were cocultured with irradiated osteoblasts to examine their therapeutic effects and mechanisms on osteoblast injury. The hUCB-MSC transplantation mouse model is used to confirm the in vivo role of hUCB-MSC treatment in radiation bone injury. Western blot analysis, qRT-PCR, immunohistochemistry, and immunofluorescence staining were used to analyze gene expression and angiogenesis. The apoptosis and migration of osteoblasts were measured by Hoechst staining, scratch test, and transwell. The differentiation of osteoblasts was measured by ALP and Alizarin red staining and transmission electron microscopy. The bone-related parameters of mice were evaluated by micro-CT analysis. We found that radiation can damage the DNA of osteoblasts; induce apoptosis; reduce the differentiation, migration, and adhesion of osteoblasts, leading to lipogenesis of bone marrow mesenchymal stem cells (BMSCs) and reducing the source of osteoblasts; and increase the number of osteoclasts in bone tissue, while MSC treatment prevents these changes. Our results reveal the inhibitory effect of radiation on osteoblast function. hUCB-MSCs can be used as a therapeutic target for the development of new therapeutic strategies for radiotherapy of bone injury diseases.

## 1. Introduction

Tumor radiation therapy is one of the common methods of treating tumors, especially malignant tumors, and radiation injury is one of the complications of radiation therapy. During radiotherapy for malignant tumors of extremities, radiotherapy for malignant tumors of the oral and maxillofacial region, and radiotherapy for bone metastasis of malignant tumors, ionizing radiation often damages the bone tissue in the treatment area [1]. On the one hand, ionizing radiation can cause pathological fractures of tumors or poor healing of bone defects after tumors [2]. On the other hand, large doses of ionizing radiation can cause bone damage in the corresponding parts, which in turn increases the risk of fractures and ultimately affects the lives of patients and even causes patients to undergo diseased bone removal and bone transplantation [3]. Numerous facts have shown that it is more difficult for patients receiving radiotherapy to heal bone defects than normal patients, including damage to the function of bone cells during radiation intervention, fibrosis, necrosis, resorption lacuna, and increase of woven lamellar bone in bone tissue [4]. The reasons for these changes may be the damage of tissue blood vessels [5, 6] and the abnormal changes in the functions of osteoblasts [7, 8], mesenchymal stem cells [9], and osteoclasts [10]. The current clinical treatment of radioactive bone injury includes surgical treatment, that is, curettage of diseased bone and bone transplantation, hyperbaric oxygen chamber treatment, and drug radiation protection treatment [4, 11]. However, these methods cannot restore the normal function of bone marrow cells, thereby repairing bone damage fundamentally. Although a variety of stem cells, including bone marrow mesenchymal stem cells (BMSCs) and adipose-derived mesenchymal stem cells (AD-MSCs), have been used for stem cell treatment of radiation-induced bone injury, their clinical access is difficult and requires invasive extraction and more complicated separation steps. Therefore, it is quite important to explore a new therapeutic modality for bone injury after radiotherapy.

Human umbilical cord blood mesenchymal stem cells (hUCB-MSC) are cells in the early stage of mesoderm development, with multidirectional differentiation potential, similar to bone marrow mesenchymal stem cells [12, 13]. It can differentiate into osteoblasts, chondrocytes, and other cells and regulate the differentiation of mononuclear macrophages under certain conditions. Therefore, these cells have been used to treat brain trauma and spinal cord injuries [14]. Transplantation of hUCB-MSC can partially improve the prognosis of traumatic rats by stimulating neurogenesis and angiogenesis and damaging the brain [15]. They are also used to alleviate the hematopoietic syndrome caused by total body irradiation [16] and articular cartilage regeneration in patients with osteoarthritis [17]. In addition, hUCB-MSCs have lower immunogenicity, which allows them to tolerate a greater degree of HLA mismatch, and have a relatively low incidence of graft-versus-host. More importantly, these cells have stronger proliferation ability and a large number of proliferation in vitro. For these reasons, they may be more ideal cells for the treatment of radiation bone injury.

Currently, there is still a lack of research on hUCB-MSCs for radiation therapy-induced bone damage. Therefore, the purpose of this research is to study the effect of hUCB-MSC on mice with radiotherapy bone injury and to explore the treatment mechanism of radiotherapy bone injury.

## 2. Material and Methods

### 2.1. Cell Culture and Osteogenic Differentiation

The second-generation human umbilical cord blood mesenchymal stem cells were purchased from Wuhan Huasai Biomedical Technology Co., Ltd., Hubei, China. The MC3T3-E1 were all derived from the American type culture collection and purchased from the BeNa Culture Collection in Suzhou. The cells were cultured in DMEM/F12 (Hyclone) with 10% fetal bovine serum (Gibco) and 1% L-glutamine at 37°C in a 5% CO_2_ atmosphere. The mouse osteogenic cell line MC3T3-E1 was cultured in DMEM (Hyclone) with 10% fetal bovine serum (Gibco) and 1% L-glutamine at 37°C in a 5% CO_2_ atmosphere; the medium was renewed every three days. When the cell fusion reached 90%, it was passaged by trypsin digestion. For osteogenic differentiation, cells were cultured with osteogenic medium (MUXMT-90021, Cyagen, Guangzhou, China) for 7 d or 14 d. Osteogenic differentiation potential was examined by the ALP assay and Alizarin red staining.

### 2.2. Model of MC3T3-E1 Cell Line

For the irradiation model, the experimental cells were plated on a 6-well plate with a density of approximately 3∗10^4^ cells/well. After plating for 24 hours, X-ray treatment was carried out with a small animal radiotherapy apparatus (X-RAD 225 Cx). The radiation dose was 6 Gy, and the time was about 4.5 minutes. The cells were then returned to the incubator to continue observation and culture. For cell coculture models, we inoculated MC3T3-E1 or hUCB-MSCs in 6-well plates or 0.4 *μ*m transwell chambers, respectively. After cocultivation for 48 h, it was used for subsequent experiments. For drug treatment, 10 *μ*M MSAB (MCE, Cat# HY-120697) was used to inhibit Wnt/*β*-catenin signaling [18].

### 2.3. RT-PCR

Total RNA was extracted by using the TRIzol Reagent (Invitrogen). The RNA was reverse transcribed using Transcriptor Universal cDNA Master (Roche) according to the manufacturer's instructions. Real-time PCR was performed as instructions. The primer sequences were as follows: caspase 3: forward (F): 5′-CATTCATGGGCCTGAAATACCAA-3′/reverse (R): 5′-CACCATGGCTTAGAATCACACACAC-3′; Runx2: F: 5′-AGATGACATCCCCATCCATC-3′/R: 5′-GTGAGGGATGAAATGCTTGG-3′; Alp: F: 5′-CACAGATTCCCAAAGCACCT-3′/R: 5′-GGTCATATAGCCGCCTCCAC-3′; OCN: F: 5′-CCTGAGTCTGACAAAGCCTTCA-3′/R: 5′-GCCGGAGTCTGTTCACTACCTT-3′; *β*-catenin: F: 5′-GCTTTCAGTTGAGCTGACCA-3′/R: 5′-AAGTCCAAGATCAGCAGTCTCA-3′; Gapdh: F: 5′-TGATGACATCAAGAAGGTGGTGAAG-3′/R: 5′-TCCTTGGAGGC CATGTAGGCCAT-3′.

### 2.4. Western Blot

The total protein of MC3T3-E1 cells was extracted with RIPA lysis buffer (Cell Signaling Technology, Cat# 9806) containing a protease inhibitor cocktail (MCE, Cat# HY-K0010, 1 : 100) and phosphatase inhibitor cocktail I (MCE, Cat# HY-K0021, 1 : 100), and the concentration was detected using the BCA kit (BCA1-1KT, Sigma, St. Louis, Missouri, USA). The protein samples were separated by 10%SDS-PAGE and then blotted onto PVDF membranes (EMD Millipore) and blocked with 5% milk. After incubating with the specific primary antibody overnight at 4°C, the blot was incubated with the corresponding horseradish peroxidase-conjugated secondary antibody for 1 hour at room temperature. Then, the ECL detection reagent (Biosharp, Cat# BL523B) was used to detect. The immunoreactive protein was detected by Tanon-5200 (Tanon, 18000856). GAPDH was used as a control. The antibodies used are Bax (Abcam, Cat# ab32503), Rad51 (Abcam, Cat# ab133534), *β*-catenin (Abcam, Cat# ab32572), Runx2 (Abcam, Cat# ab236639), and Gapdh (Proteintech Group In, Cat# 60004-1-Ig).

### 2.5. Apoptosis and Migration Detection

For apoptosis detection, cells were taken at 48 hours, washed with PBS buffer for 3 times, and fixed with paraformaldehyde fixative for 15 minutes. After fixation, wash properly to remove the fixative, add a small amount of Hoechst (Beyotime, Cat# C1017) staining solution to the adherent cells, and leave at room temperature for 3-5 minutes. Aspirate the staining solution and wash 3 times with TBST solution for 5 minutes each time. Immediately observe and take pictures under an inverted fluorescence microscope; the excitation wavelength is about 346 nm.

For the horizontal migration ability, cells were subjected to a scratch test. A 10 *μ*L pipette tip was used to draw regular “cross”-shaped cell scars on the culture plates of each group and then photographed. The cells were taken every 2 hours. Observe and take pictures until the cell scratches are healed.

For the spatial migration ability test, pour about 2∗10^4^ cells/well in the upper chamber of a transwell six-well plate, and add 1 mL of serum-free medium. In the lower layer of the transwell six-well plate, add 2 mL of complete medium and place it in the incubator for cell culture. After 24 hours, the culture medium was aspirated and fixed with paraformaldehyde, and 0.1% gentian violet staining solution was added for staining after washing. After dyeing for 10 minutes, remove the gentian violet staining solution, wash with PBS until the well plate chamber is clean, and stand still at room temperature overnight. After overnight, after the transwell six-well plate cell is dry, the cell is observed and photographed.

### 2.6. Animal Experiments

The study was approved by the Ethics Committee of Wuhan University. The experimental animal model used 6-week-old C57BL/6 inbred male mice, purchased from Beijing Weitonglihua Experimental Animal Technology Co., Ltd. After several days of adaptive culture, the experimental mice were divided into three groups: A, B, and C. Group A was the control group, group B was radiotherapy alone, and group C was the radiotherapy+injection of human umbilical cord blood mesenchymal stem cell group. Each mouse in group B and group C was subjected to X-ray whole-body irradiation treatment by small animal radiotherapy apparatus, the irradiation dose was 6 Gy, and the time was about 4.5 minutes. After the irradiation, 300 *μ*L hUCB-MSCs with a concentration of 1∗10^7^/mL were injected into the tail vein of each mouse in group C, and 300 *μ*L normal saline was injected into the tail vein of each experimental mouse in groups A and B. On the 28th day, all the experimental mice were sacrificed to obtain materials, one side of the humerus was put into the electron microscope fixation solution, and the other side of the humerus was put into the paraformaldehyde fixation.

### 2.7. Micro-CT Analyses

Place the humerus on the scanner of the SkyScan 1176-high-resolution in vivo MicroCT instrument for scanning, and the scanning thickness is 9 mm. After scanning the samples of mice in groups A, B, and C, reconstruct the pictures, and then analyze the data, BV/TV, Conn.D, Tb.N, Tb.sh, and Tb.Th. After the analysis is completed, the expression difference is detected.

### 2.8. Transmission Electron Microscope (TEM)

The mouse bone tissue was decalcified for 20 days and fixed with 2.5% glutaraldehyde in 0.1 M sodium dihydrogen phosphate (pH 7.4) at 2.5°C for 4 hours, and then, the samples were fixed with 1% OsO_4_ for 1 hour at room temperature. The specimen was then dehydrated and infiltrated. The sections were stained with uranyl acetate and lead citrate. A transmission electron microscope (Hitachi, HT-7800) was used to observe mouse bone tissue.

### 2.9. Histological Analyses

For paraffin sections, the humerus was fixed in 4% paraformaldehyde for 72 hours, decalcified in 10% EDTA for 20 days, and then embedded in paraffin. They were cut into 8 *μ*m thickness with Leica RM2235 microtome and stained with TRAP and HE after deparaffinization. *α*-SMA (Abcam, Cat# ab7817) was used to label the vascular smooth muscle and perform immunofluorescence detection.

### 2.10. Immunohistochemistry

Immunohistochemical analysis was performed on the samples to observe genetic changes associated with mouse angiogenesis. First, the sections were deparaffinized, and an antigen search was performed using citrate buffer (pH 6.0) at 95°C. The samples were then treated with 3% H_2_O_2_ at room temperature for 20 minutes and blocked with 5% BSA at 37°C for 1 hour at room temperature. VEGF antibody (Servicebio, Cat# GB11034B) was used for immunohistochemical staining and incubated with sections overnight at 4°C. After washing, the Polink-2 Plus Polymer HRP detection system (ZSGB-BIO, Cat# PV6001) and DAB (ZSGB-BIO, Cat# ZLI-9017) were used for colour development according to the manufacturer's instructions. Hematoxylin was used for nuclear staining. The sections were then dehydrated, fixed, and scanned with Aperio VERSA 8.

### 2.11. Statistical Analysis

All data is represented as the mean ± standard deviation (SD). Statistical differences between the two groups were evaluated by one-way analysis of variance, and Tukey tests were performed as needed. All experiments were repeated at least 3 times, and *P* < 0.05 was considered significant.

## 3. Results

### 3.1. Irradiation Inhibits the Function of Osteoblast Progenitor Cells

**Figure 1:**
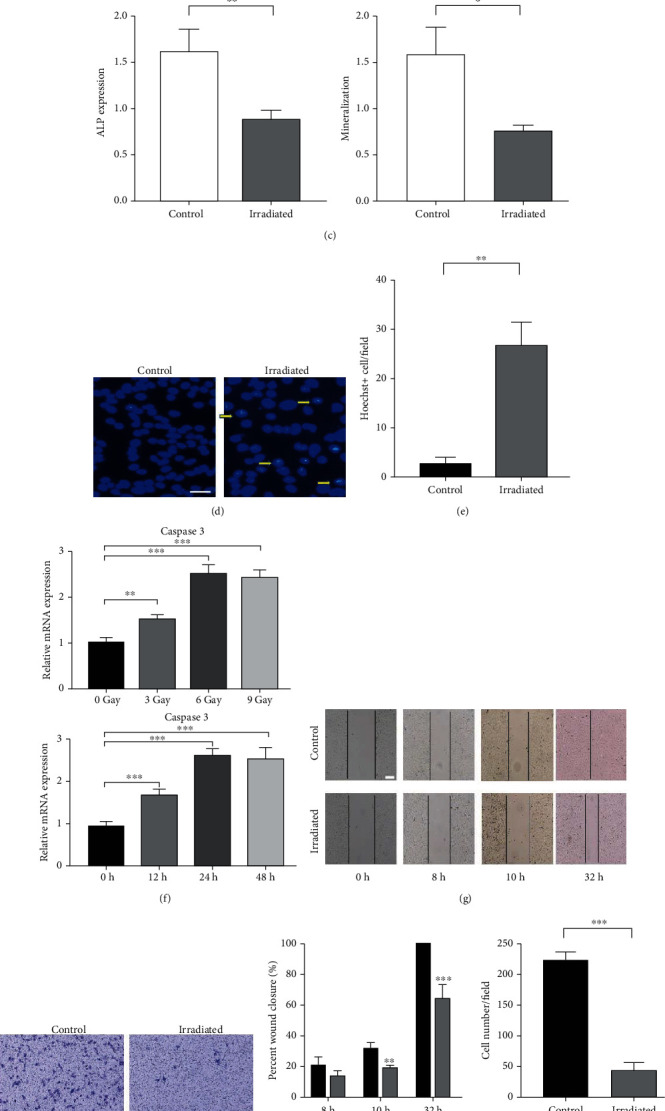
Irradiation inhibits the function of osteoblast progenitor cells. (a) ALP staining in control and irradiated MC3T3 (scale bar, 100 *μ*m). (b) Alizarin red staining in control and irradiated MC3T3. (c) Statistics of ALP and Alizarin red in control and irradiated MC3T3. (d) Hoechst staining in control and irradiated MC3T3. The yellow arrowhead points to apoptotic cells (scale bar, 25 *μ*m). (e) Statistics of Hoechst in control and irradiated MC3T3. (f) Apoptosis-specific gene expression in different times and doses with X-ray cells. (g) Scratch test in control and irradiated MC3T3. (h) Transwell in control and irradiated MC3T3 (scale bar, 100 *μ*m). (i) Statistics of scratch and transwell in control and irradiated MC3T3. All data are mean ± SD; ^∗^*P* < 0.05, ^∗∗^*P* < 0.01, and ^∗∗∗^*P* < 0.001.

In order to explore the damage effect of radiotherapy on bone, we induced osteogenic differentiation of MC3T3-E1 cell lines treated with 0 Gy or 6 Gy irradiation. MC3T3-E1 cells irradiated with 6 Gy had a significantly lower ALP staining positive rate (Figures 1(a) and 1(c)). Alizarin red staining showed that calcium nodules and scattered calcium salts were also significantly reduced compared to the 0 Gy group (Figures 1(b) and 1(c)). It can be seen that high-dose irradiation can reduce the osteogenic differentiation ability of osteoblast precursor cells. After that, MC3T3-E1 cells treated with 0 Gy or 6 Gy irradiation were cultured for 48 hours, and the cells of each group were stained with Hoechst and quantitatively analyzed. The results showed that the MC3T3-E1 cells in the irradiated group had more Hoechst-positive cells than the control group, and the cell morphology has also undergone great changes (Figures 1(d) and 1(e)). After irradiation, some MC3T3-E1 cells became larger, swelled and are irregular, and tended to apoptosis. At the same time, the apoptosis-related gene caspase 3 of the irradiated osteoblast precursor cells was significantly increased (Figure 1(f)). It can be seen that single high-dose radiation can cause apoptosis of some osteogenic precursor cells.

In order to explore the effect of irradiation on the migration ability of osteoblast precursor cells, we cultured MC3T3-E1 cells treated with 0 Gy or 6 Gy for 5 days. The scratch healing test and transwell migration test were performed on each group of cells, and quantitative analysis was performed. We found that the difference in scratch healing between the irradiated group and the control group was observed at 8 h after scratching, but there was no statistical difference. The scratch healing ability of the irradiated group was significantly lower than that of the control group from 10 h. At 32 h, the scratches in the control group were completely healed, while the irradiated group was still in an unhealed state (Figure 1(g)). Meanwhile, for the transwell migration experiment, we observed that the number of MC3T3-E1 cells in the irradiated group that migrated below the transwell cell was significantly less than that of the control group after 24 hours of migration under an 8 *μ*m pore size (Figures 1(h) and 1(i)). It can be seen that after a single high-dose irradiation treatment of osteoblast precursor cells, their horizontal migration ability and three-dimensional space migration ability are significantly reduced. To sum up, the function of osteoblast precursor cells is significantly inhibited after radiation.

### 3.2. hUCB-MSCs Can Rescue the Function of Irradiated Osteogenic Precursor Cells

**Figure 2:**
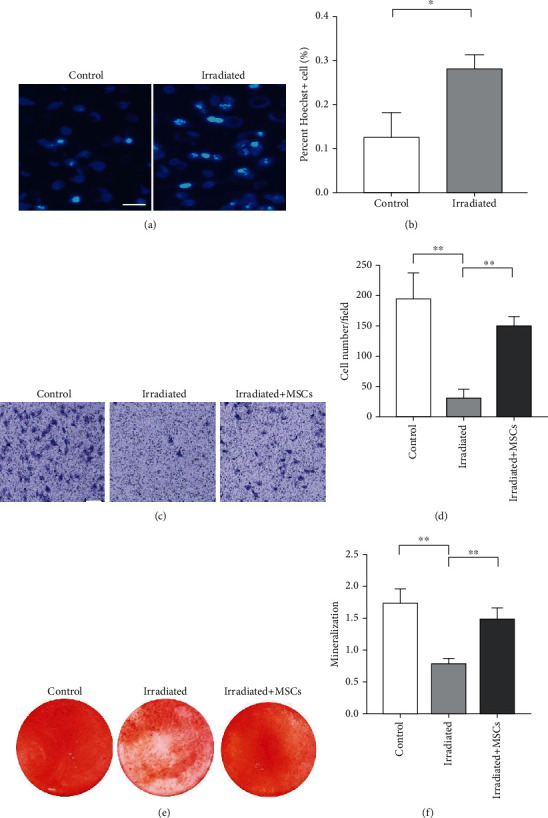
hUCB-MSCs can rescue the function of irradiated osteogenic precursor cells. (a) Hoechst staining in MSC treatment and irradiated MC3T3 (scale bar, 100 *μ*m). (b) Statistics of Hoechst in MSC treatment and irradiated MC3T3s. (c) Transwell in control, MSC treatment, and irradiated MC3T3 (scale bar, 100 *μ*m). (d) Statistics of transwell in control, MSC treatment, and irradiated MC3T3. (e) Alizarin red staining in control, irradiated, and irradiated with MSC treatment MC3T3. (f) Statistics of Alizarin red staining in control, irradiated, and irradiated with MSC treatment MC3T3. All data are the mean ± SD; ^∗^*P* < 0.05;  ^∗∗^*P* < 0.01.

In order to study the rescue effect of hUCB-MSCs on irradiated osteoblast precursor cells, we treated MC3T3-E1 with 0 Gy or 6 Gy ionizing radiation and immediately cocultured with hUCB-MSCs. After 48 h, MC3T3-E1 cells were performed apoptosis-related detection and quantitative analysis. The results showed that the Hoechst-positive cells increased in the osteoblasts treated with coculture of hUCB-MSCs instead of decreasing (Figure 2(a)), and the Hoechst-positive rate was significantly different between the two groups (Figure 2(b)). It indicated that the coculture treatment of hUCB-MSCs promoted the apoptosis of damaged osteoblasts rather than reversing the trend of osteoblast apoptosis. After that, we divided the osteoblast precursor cells into the irradiation group, the hUCB-MSC treatment group, and the control group. After 48 hours of coculture, we continued to culture them for 3 days. Then, the migration ability of each group of cells was detected through the transwell migration experiment. The result showed that the number of cells in the treatment group was significantly different from that of the irradiation group (Figure 2(c)). Although the irradiated MC3T3-E1 cells failed to recover to the normal migration ability of the control group after coculture treatment with hUCB-MSCs, their migration and homing ability had recovered to about 70% (Figure 2(d)). In addition, we found that MC3T3-E1 cells treated with hUCB-MSCs had a higher positive rate of calcium deposit formation than the irradiation group (Figures 2(e) and 2(f)), which indicated that the hUCB-MSC treatment reversed the damage of mineralization caused by irradiation. These results show that hUCB-MSCs can reverse the osteogenic differentiation ability damaged by radiation.

### 3.3. hUCB-MSCs Regulate Osteogenic Differentiation of Damaged Cells through Wnt/*β*-Catenin

**Figure 3:**
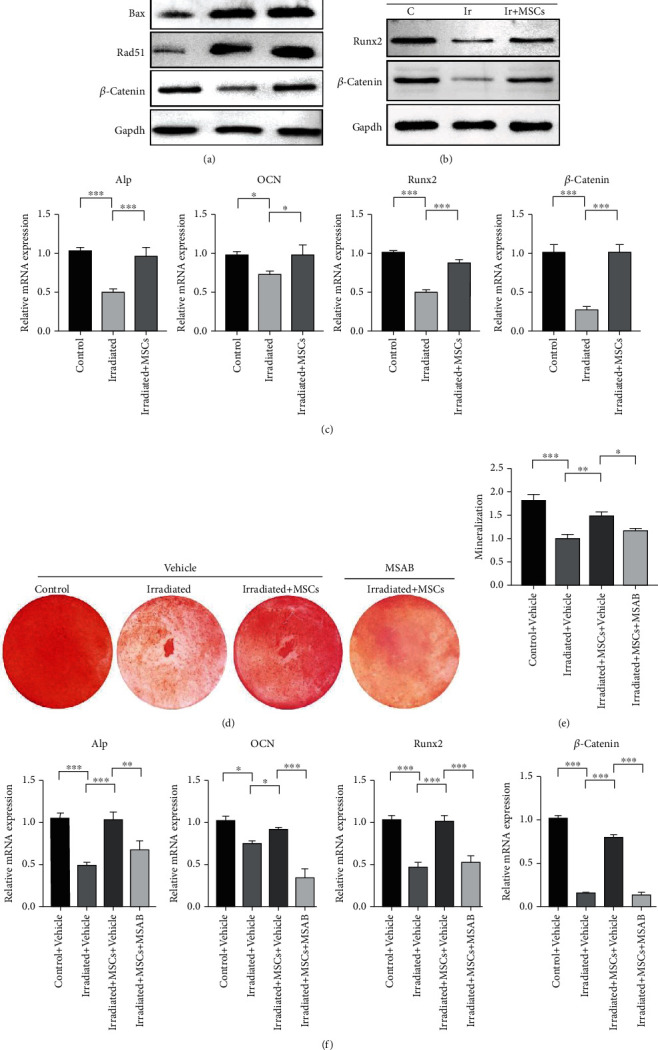
hUCB-MSCs can regulate osteogenic differentiation of damaged cells through Wnt/*β*-catenin. (a) Western blot to detect the expression of apoptosis markers in control, MSC treatment, and irradiated MC3T3. (b) Western blot to detect the expression of osteogenic differentiation markers in control, MSC treatment, and irradiated MC3T3. (c) The expression of osteogenic differentiation-specific genes in MC3T3 of control, irradiated, and irradiated with MSC treatment. (d) Alizarin red staining in control, irradiated, irradiated with MSCs, and irradiated with MSCs and MSAB MC3T3. (e) Statistics of Alizarin red staining in control, irradiated, irradiated with MSCs, and irradiated with MSCs and MSAB MC3T3. (f) The expression of osteogenic differentiation-specific genes in MC3T3 of control, irradiated, irradiated with MSCs, and irradiated with MSCs and MSAB. All data are mean ± SD; ^∗^*P* < 0.05,  ^∗∗^*P* < 0.01, and^∗∗∗^*P* < 0.001.

We divided the cells into an irradiation group, a treatment group, and a control group as previously described. RNA and protein were isolated for detection at 0 and 8 days of osteogenic differentiation. The results showed that the expression of Bax in the irradiation group was significantly higher than that in the control group. The expression of Bax in the treatment group was higher than that in the irradiation group, which was consistent with the previous experimental results. hUCB-MSCs can promote the apoptosis of irradiated MC3T3-E1 cells. Rad51 expression in the irradiation group was significantly higher than that in the control group, indicating that radiation activated the DNA homologous recombination repair-related genes of MC3T3-E1, while the expression of Rad51 in the treatment group was higher than that in the irradiation group (Figure 3(a)). It showed that hUCB-MSC coculture can promote DNA homologous recombination repair of MC3T3-E1 cells, reduce DNA double-strand breaks, and repair cell damage caused by irradiation. Moreover, the expression of RUNX2 and *β*-catenin protein in the irradiation group was significantly lower than that in the control group, while coculture of hUCB-MSCs could rescue the expression of *β*-catenin in irradiated MC3T3-E1 cells (Figure 3(b)). It showed that hUCB-MSCs may reverse the migration and osteogenic differentiation of damaged cells by regulating Wnt/*β*-catenin. Indeed, the detection of qPCR also showed a similar result (Figure 3(c)). To examine the role of Wnt/*β*-catenin in the effect of hUCB-MSCs on osteogenic differentiation, MSAB (a selective inhibitor of Wnt/*β*-catenin signaling) was used to specifically inhibit Wnt/*β*-catenin signaling. As shown in the figure, the irradiation treated with the hUCB-MSCs and MSAB groups had a lower positive rate of calcium deposit formation than irradiation treated with the hUCB-MSC group (Figures 3(d) and 3(e)). Consistently, MSAB-treated MC3T3 exhibited downregulated expression of osteogenesis differentiation genes, including OCN, Runx2, Alp, and *β*-catenin (Figure 3(f)). These results indicated that MSAB treatment inhibited the rescue effect of hUCB-MSCs on osteogenic differentiation and hUCB-MSCs can reverse the osteogenic differentiation of irradiated cells through Wnt/*β*-catenin signaling.

### 3.4. hUCB-MSC Treatment Can Rescue the Effect of Irradiation on Bone Mass in Mice

**Figure 4:**
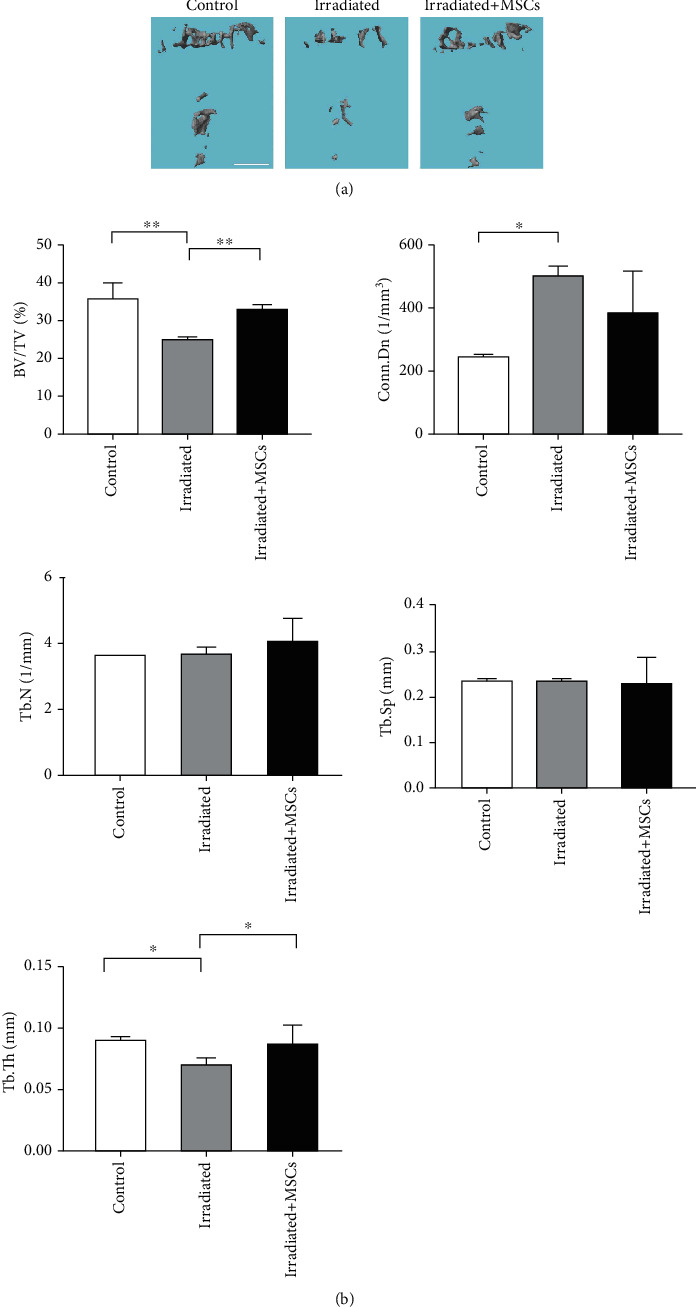
hUCB-MSC treatment can rescue the effect of irradiation on bone mass in mice. (a) Representative *μ*CT images (scale bars, 0.5 mm). (b) Statistics of cortical bone parameters by *μ*CT from mice implanted with control, MSC treatment, and irradiated (*n* = 5). Tb.Sp; Tb.N; Tb.Th; BV/TV; Conn.Dn. All data are mean ± SD; ^∗^*P* < 0.05;  ^∗∗^*P* < 0.01.

To verify the therapeutic effect of hUCB-MSCs in vivo, we divided the mice into the irradiation group, the irradiation treatment with hUCB-MSC group, and the control group. After 6 Gy of irradiation treatment for the irradiation group and irradiation treatment with the hUCB-MSC group, the hUCB-MSC treatment group was treated with intravenous injection of hUCB-MSCs immediately, and the other two groups were injected with the same amount of normal saline. On the fourth day, human-derived mesenchymal stem cells were detected in the peripheral blood of the mice, proving the success of stem cell transplantation. After the mice were raised for 28 days, the forelimbs of the mice were tested by micro-CT, and the test data were quantitatively analyzed. The results showed that the bone mass of the irradiated mice was significantly lower than that of the control group, while the bone mass of the mice treated with hUCB-MSCs was significantly higher than that of the irradiated group. Although it was less than the bone mass of the control group, it was already close to the bone mass of the control mice (Figure 4(a)). The BV/TV and Tb.Th of the irradiated group were significantly lower than those of the control group (Figure 4(b)), while the BV/TV and Tb.Th of the treatment group were significantly higher than that of the irradiated group (Figure 4(b)). It indicated that intravenous injection of hUCB-MSCs can reverse the bone loss of irradiated mice at a dose of 6 Gy and reduce the risk of pathological fractures.

### 3.5. hUCB-MSCs Reverse Bone Loss by Improving the Bone Marrow Microenvironment

**Figure 5:**
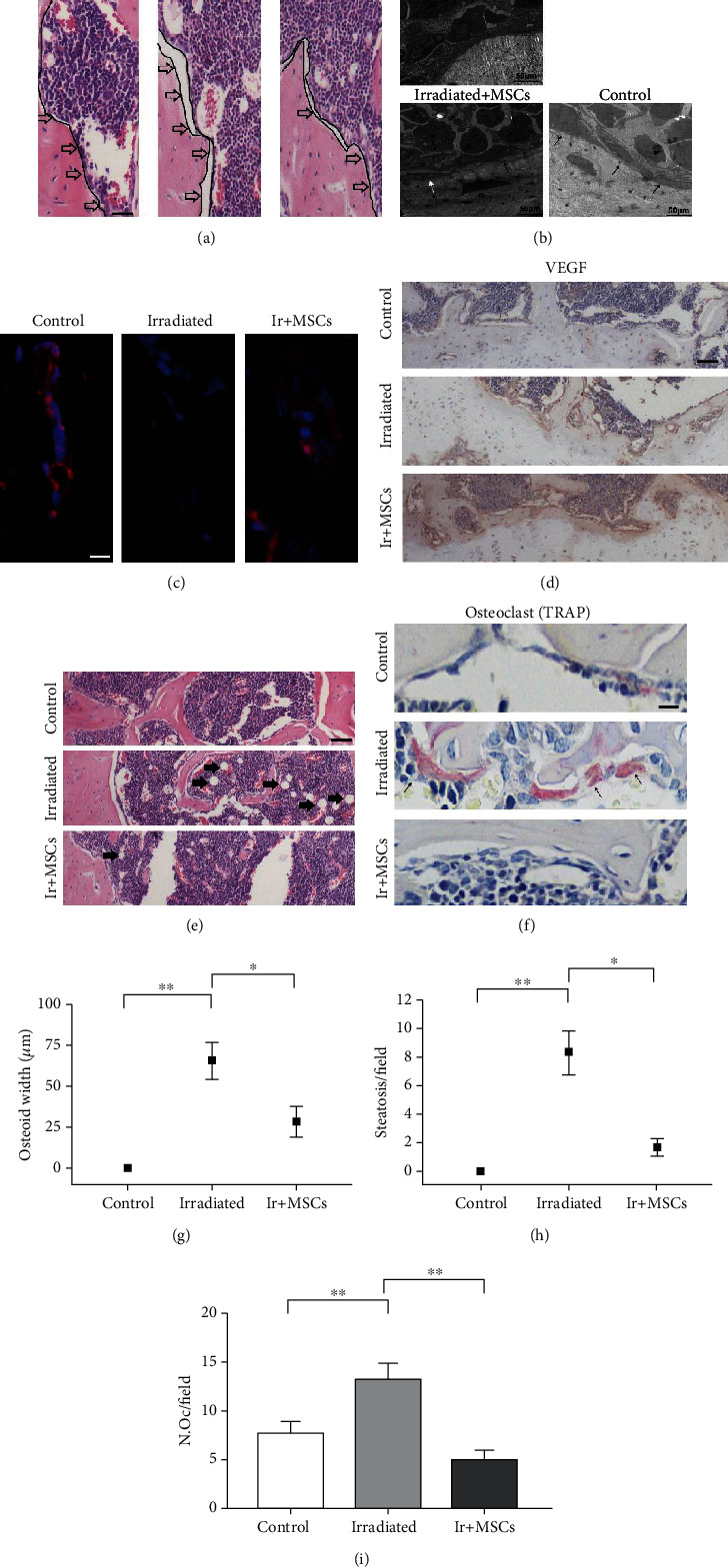
hUCB-MSCs can save bone loss by improving the bone marrow microenvironment. (a) H&E of humerus sections from mice with control, MSC treatment, and irradiated. The black line showed the distance between the bone surface and the adjacent bone marrow tissue (scale bar, 100 *μ*m). (b) Transmission electron microscopy images of humerus sections from mice with control, MSC treatment, and irradiated. The black arrowhead points to osteoprogenitor cells. The white arrowhead points to immature osteocytes and osteogenic phenomena (scale bar, 50 *μ*m). (c) *α*-SMA staining of humerus sections from mice with control, MSC treatment, and irradiated (scale bar, 25 *μ*m). (d) Immunohistochemistry of humerus sections from mice with control, MSC treatment, and irradiated (scale bar, 250 *μ*m). (e) H&E of humerus sections from mice with control, MSC treatment, and irradiated. The black arrowhead points to fat cells (scale bar, 250 *μ*m). (f) TRAP staining of humerus sections from mice with control, MSC treatment, and irradiated. The black arrowhead points to osteoclasts (scale bar, 25 *μ*m). (g) Statistics of osteoid width in the control, MSC treatment, and irradiated groups. (h) Statistics of fat particles in the control, MSC treatment, and irradiated groups. (i) Statistics of osteoclasts in the control, MSC treatment, and irradiated groups. All data are mean ± SD; ^∗^*P* < 0.05;  ^∗∗^*P* < 0.01.

The humerus of the above three groups of mice was taken for related detection. We found that the distance between bone surface and adjacent bone marrow tissue in the irradiation group was significantly wider than that in the control group, while the distance between bone surface and adjacent bone marrow tissue in hUCB-MSC-treated mice was restored. Although it cannot be restored to the level of the control group, the distance between bone surface and adjacent bone marrow tissue was smaller than that in the irradiated group and similar to the control group (Figures 5(a) and 5(g)). Meanwhile, transmission electron microscopy also showed that a layer of regular and continuous osteoblasts or osteoprogenitor cells formed at the junction of bone and bone marrow tissue (especially cancellous bone) in the control group, while osteoblast progenitor cells in the irradiated group were damaged. Compared with the control group, the osteoprogenitor cells were not arranged continuously, tightly, and orderly on the bone surface in the irradiated group. In contrast, the mice in the treatment group showed regular, tight, and orderly arrangement of osteoprogenitor cells at the bone marrow tissue junction, which was similar to the control group (Figure 5(b)). Moreover, new osteogenesis was observed in the treatment group (Figure 5(b)). It also showed that the osteogenic precursor cells after hUCB-MSC treatment had restored their osteogenic activity. Then, the small arterial blood vessels in the bone tissue of the irradiated group were significantly reduced compared with the control group and the hUCB-MSC treatment group, while the bone tissue of the mice treated with hUCB-MSCs had new small blood vessels, which was similar to the control group (Figure 5(c)). And the expression of vascular endothelial growth factor (VEGF) was observed to increase significantly compared with the control group and the irradiation group (Figure 5(d)). It showed that intravenous injection of hUCB-MSCs may promote the regeneration of damaged tiny blood vessels in bone tissue, thereby restoring the blood supply of bone tissue and providing favorable conditions for the repair of bone injury. Furthermore, a large number of fat particles appeared in the bone marrow tissue of the irradiated group. In the bone marrow tissue treated with hUCB-MSCs, fatty bone marrow tissue still exists, but the content was less (Figures 5(e) and 5(h)). The treatment group also inhibited the activation of osteoclasts caused by irradiation, and the number was similar to that of the control group (Figures 5(f) and 5(i)). These results prove that hUCB-MSCs can fundamentally improve the bone destruction caused by irradiation *in vivo*, and it is expected to become a new treatment method for bone destruction after radiotherapy.

## 4. Discussion

Radiation bone injury is a common clinical complication; especially for patients undergoing supplemental radiotherapy during and after bone tumor surgery, the bone tissue around the lesion is difficult to heal and even causes osteonecrosis and infection [19]. The main reasons for radioactive bone injury include the vascular damage of bone tissue and bone marrow tissue caused by radiation [20], which depletes the mesenchymal stem cells in the bone marrow, thereby reducing the source of osteoblasts or directly affecting the function of osteoblasts. Radiation can also increase the differentiation of osteoclasts, break the balance between osteoblasts and osteoclasts, and aggravate bone loss [21, 22]. In this study, we found that the irradiated osteoblast precursor cells showed lower osteogenic differentiation ability, lower migration ability, and higher apoptosis level, which indicated that a single large dose of irradiation can affect various functions of osteoblast precursor cells.

Transplantation of mesenchymal stem cells locally or systemically is one of the effective treatments for radiation injury [23, 24]. Three main types of MSCs are used for cell therapy, including BMSCs, AD-MSCs, and UCB-MSCs [25]. However, BMSCs and AD-MSCs are difficult to obtain clinically and require invasive extraction or complicated separation steps. UCB-MSCs are cells with the characteristics of totipotent stem cells in the early stage of mesoderm development [26]. They can differentiate into osteoblasts, chondrocytes, and skeletal muscle cells with lower immunogenicity. More importantly, UCB-MSCs have stronger proliferation ability and are more ideal cells for the treatment of radiation bone injury. When conducting cell experiments in vitro, we found that stem cell therapy promoted the apoptosis of irradiated cells instead of improving the apoptosis of osteoblasts. The expression of Bax can also support this phenomenon. This may be a positive regulation, because some osteoblasts are unable to necrotize and apoptose in time and lose their differentiation function after radiation. The addition of hUCB-MSCs promoted programmed death of injured cells and retained osteoblasts with normal differentiation and migration functions. This was verified by subsequent migration and osteogenic differentiation experiments. hUCB-MSC treatment can not only improve the migration and homing ability of osteoblasts but also greatly enhance the osteogenic mineralization ability of osteoblasts.

When the cell is exposed to radiation, the double-stranded and single-stranded DNA will break, which will initiate the DNA repair program in the cell [27, 28]. Rad51 is the key protein of this pathway [29]. In our experiments, the Rad51 protein of the irradiated group was significantly higher than that of the control group, which means that radiation induced homologous recombination repair of osteoblasts, and hUCB-MSCs can increase the expression of Rad51 protein in the irradiated cells. It showed that hUCB-MSCs can further activate the homologous recombination repair signaling pathway of osteoblasts and promote the repair of broken double-stranded DNA, thereby improving the DNA integrity of osteoblasts and ultimately retaining various biological functions of osteoblasts.

Finally, we established an irradiated animal model to verify the therapeutic effect of hUCB-MSCs. The irradiated mice showed significant bone loss, which was mainly manifested as the decrease of BV/TV and Tb.Th. In contrast, BV/TV and Tb.Th were recovered in mice receiving hUCB-MSC transplantation. Therefore, hUCB-MSC transplantation is effective in treating radiation-induced bone injury.

In order to explore the mechanism of hUCB-MSC treatment, we analyzed the bone tissue of mice and found the treatment group had more blood vessels and higher VEGF expression than the irradiation group, indicating that hUCB-MSCs can promote bone tissue angiogenesis, establish a good internal environment for bone repair, and achieve the therapeutic effect of radiation bone injury. Meanwhile, the treatment group can reduce the increase in “osteoid width” caused by irradiation, which is generally considered to be the distance between bone and adjacent bone marrow tissue and related to the decrease in the number of osteoprogenitor cells or adhesion function. It pointed out that radiation can reduce the number of bone progenitor cells or decrease the adhesion function in bone tissue, and hUCB-MSC transplantation can largely reverse these changes. Moreover, the treatment group also had fewer fat cells and osteoclasts than the irradiation group, indicating that the treatment of hUCB-MSCs reestablished the homeostasis of the bone marrow environment.

## 5. Conclusions

In summary, our research showed that hUCB-MSCs can improve the blood vessels of irradiated mice, improve the migration, adhesion, and osteogenic differentiation of osteoblasts, and reduce the activation of osteoclasts and the fatification of bone marrow mesenchymal stem cells, thereby restoring the balance of osteogenesis and osteoclastogenesis, reducing bone loss, and finally achieving the purpose of curing radiation bone injury.

## Data Availability

The datasets used and/or analyzed during the current study are available from the corresponding authors on reasonable request.

## Abbreviations

RIBI: Radiation-induced bone injury
BMSCs: Bone marrow mesenchymal stem cells
AD-MSCs: Adipose-derived mesenchymal stem cells
hUCB-MSCs: Human umbilical cord blood mesenchymal stem cells
TEM: Transmission electron microscopy
VEGF: Vascular endothelial growth factor
MSCs: Mesenchymal stem cells.
